# Duodenal Bulb Mucosa with Hypertrophic Gastric Oxyntic Heterotopia in Patients with Zollinger Ellison Syndrome

**DOI:** 10.1155/2009/298381

**Published:** 2009-07-01

**Authors:** Emil Kohan, David Oh, Hank Wang, Salar Hazany, Gordon Ohning, Joseph R. Pisegna

**Affiliations:** ^1^CURE: VA/UCLA Digestive Disease Research Center, Department of Medicine, David Geffen School of Medicine at University of California Los Angeles, Los Angeles, CA 90024, USA; ^2^Division of Gastroenterology and Hepatology, VA Greater Los Angeles Healthcare System, 11301 Wilshire Blvd, Los Angeles, CA 90073, USA

## Abstract

*Objectives*. Zollinger-Ellison Syndrome (ZES) results in hypersecretion of gastric acid (via gastrinoma) leading to peptic ulcers, diarrhea, and abdominal pain. We describe the novel discovery of hypertrophic, heterotopic gastric mucosa in the proximal duodenal bulb in patients with ZES, which we hypothesize results in an increased incidence of postbulbar ulcers in patients with ZES (a mechanism previously unreported). We determined the incidence of the novel finding of duodenal gastric oxyntic hypertrophic heterotopia (GOH) in patients with ZES. *Methods*. Seven patients with ZES were enrolled. The diagnosis of ZES was established by hypergastrinemia, gastric acid hypersecretion, and a positive secretin test or based on biopsy specimens (evaluated via tissue staining). Basal acid output (BAO) and baseline gastrin secretion were determined by established methods. Endoscopic examinations with methylene blue staining and biopsy of the gastric and duodenal mucosa were conducted in all patients every 3–6 months for an average of 5 years. *Results*. The duodenal mucosa demonstrated hypertrophic GOH in 5 out of 7 patients with ZES and an intact stomach and duodenum. Biopsies from the bowel mucosa demonstrated patchy replacement of surface epithelium by gastric-type epithelium with hypertrophic oxyntic glands in the lamina propria in 5 patients. Two of the patients had no evidence of GOH in the duodenal bulb. Patients with GOH had an average serum gastrin level of 1245 pg/mL and BAO of 2.92 mEq/hr versus 724 pg/mL and 0.8 mEq/hr in patients without GOH. *Conclusions*. This study demonstrated the presence of duodenal mucosa with GOH in 5 out of 7 patients with ZES and an intact stomach and duodenum. The presence of hypertrophic and heterotopic gastric mucosa is proposed to result from increased gastrin levels and may contribute to the increased incidence of postbulbar ulcers in these patients.

## 1. Introduction

Zollinger Ellison Syndrome (ZES) is a rare gastrointestinal syndrome that results from a gastrin-producing tumor generally localized to either the pancreas or duodenum [1]. The pathophysiological state resulting from ZES is attributable to excessive release of gastrin and the consequent effects of hypergastrinemia [1]. A substantial secretion of acid results in damage to the mucosal lining of the GI tract. Acid-associated severe and recurrent gastrointestinal ulcerations with a high incidence of bleeding and perforation make gastric acid hypersecretion a major cause of morbidity and mortality in patients with ZES. Additionally, patients with ZES can develop esophagitis, duodenojejunitis, maldigestion, and diarrhea that are often debilitating and adversely affect their quality of life [2]. In addition to the effects on gastric acid secretion, gastrin stimulates gastric mucosal proliferation [3–5]. The effect of gastrin on gastric mucosa proliferation has been well explained and accounts for the hyperplasia and hypertrophy of the parietal cell, which has been illustrated in patients with ZES [6].

Heterotopic gastric tissue has been described as a congenital lesion in the duodenal bulb in between 0.5–2% of the general population [7–10]. Mann et al. provide an excellent description of heterotopic gastric tissue (GOH) in the duodenal bulb and elucidate the differences between heterotopic gastric tissue and gastric metaplasia (GM) [11]. It is important that GOH and GM are correctly differentiated because of their different clinical manifestations. GOH is histopathologically and clinically different from the much more common duodenal metaplasia. These two lesions have historically been mistaken as one and the same [11–14]. However the importance of differentiating between them is underscored in the background of ZES. It is important that GOH and GM are correctly differentiated because of their different clinical manifestations. GOH is benign, described as a macroscopic lesion on endoscopic examination with masses up to 2.5 cm or multiple polyps up to 10 mm each. On histology, these lesions demonstrate well-differentiated, well-organized gastric glands with mature structures including chief cells, parietal cells, and mucus cells. GOH cannot be diagnosed in the presence of *H. pylori*. GM is always acquired, seen as a microscopic lesion on endoscopy, and often demonstrates mucus neck cells with occasional parietal cells [11, 15, 16].

In this report, we describe the endoscopic and histopathological characteristics of the novel finding of hypertrophic, heterotopic duodenal gastric mucosa in patients with Zollinger Ellison syndrome. The discovery of heterotopic gastric mucosa within the duodenal bulb during endoscopic biopsy is a relatively recent discovery, due in part to the use of more slender endoscopes permitting retroflexion within the duodenal bulb. This permits a full examination of the duodenal bulb. Given that the majority, if not all, of the patients with ZES have gastric mucosal hyperplasia, we hoped that investigation of this group of patients for the presence of heterotopic gastric mucosa in the duodenal bulb would reveal unique, clinically relevant findings. Although GOH is a benign finding in the general population, duodenal hypertrophic heterotopic gastric mucosa may contribute to postbulbar ulcerations commonly seen in patients with ZES. An appreciation for this can also be used to consider the diagnosis of ZES prior to gastric acid secretory studies.

## 2. Materials and Methods

### 2.1. Patients

Seven subjects were enrolled in the study to be evaluated for GOH. They were diagnosed with Zollinger Ellison Syndrome using the following established criteria: elevated serum gastrin (>1000 pg/mL) in the presence of gastric acid secretion (>15 mEq/hr or >5 mEq/hr if the patient had previous gastric surgery) as well as a positive provocative testing with secretin stimulation (a rise in serum gastrin ≥200 pg/mL after injection) or with calcium infusion (a rise in serum gastrin ≥395 pg/mL), a positive histological diagnosis of gastrinoma, or a combination of these [17]. All subjects were over 18 years of age, and female subjects were not pregnant and either postmenopausal or using medically acceptable contraception. All patients presented with abdominal symptoms, including pain and diarrhea, at the time of diagnosis. Each subject had a comprehensive history taken and underwent a complete physical examination. The prescreening evaluation included a complete blood cell count, chemistry, liver function tests, gastrin, thyroid function tests, pregnancy test (for premenopausal women only), and urinalysis. During the study, vital signs and EKGs were also monitored, and laboratory evaluations were obtained at specified times.

The patients were enrolled with a mean age of 49.6 years (range of 38–61 years). Five of the 7 patients were males, and two females (Table 1). The average time since the diagnosis of ZES was 11.3 years (range of 1–25 years). Multiple endocrine neoplasia type I (MEN I) was diagnosed in 3 of the patients. None of the seven patients had been cured of ZES at the time of study enrollment and none had received chemotherapy prior to study enrollment. All of the patients were treated with pantoprazole with an average dose of approximately 90 mg daily (range of 80–160).

### 2.2. Endoscopic Evaluation

Patients underwent endoscopic evaluation with a Pentax 2700 or 2900 endoscope following intravenous sedation with midazolam and meperidine. The endoscope was advanced to the duodenal bulb, inspection was performed, and photography taken. Alcian blue was used to highlight the contours of the gastric mucosa in the duodenal bulb. Biopsies and photographs were taken with the scope in the retroflexed position. Biopsies were mounted and oriented and placed in formalin fixative. Patients underwent endoscopies at 3–6 month intervals for over a period of four to five years to monitor any changes related to disease and the effect of PPI treatment.

### 2.3. Study Design

Subjects were evaluated for GOH as described in “endoscopic evaluation.” The acid output (AO) was determined as described previously [18]. The value of AO was expressed in mEq/hr and calculated by determining the total acid in four 15-minute gastric collections utilizing a nasogastric tube. The tube was passed into the dependent portion of the stomach, and its position was verified by the water recovery method [18]. After aspiration of the residual gastric content for approximately 15 minutes, basal acid secretion was collected in 15-minute intervals for one hour using low intermittent suction. Gastric aspirates were assayed for total acid content by titration with 0.1 M NaOH to pH 7.0 at the time of collection.

## 3. Results

These demographic and gastric secretory data are consistent with the majority of patients who are diagnosed with ZES and who are under treatment with proton pump inhibitors ([17]—mentioned before). Diarrhea was the most common symptom, followed by abdominal pain and nausea, for subjects involved in this study.

**Figure 1:**
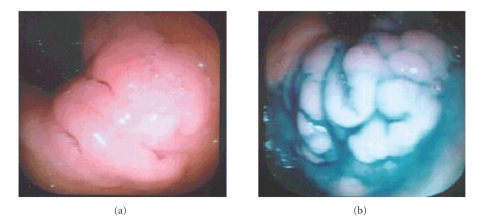
Heterotopic Gastric Mucosa in the Duodenum unstained (a) and with Methylene Blue-stained Duodenal tissue. (b) These lesions were seen as macroscopic lesions on endoscopy.

**Figure 2:**
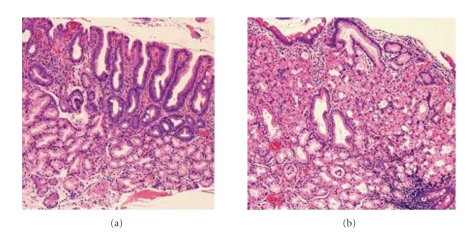
Microscopic images of endoscopic biopsy of the duodenum in patients with and without hypertrophic GOH. The relatively normal mucosa in patients and areas not affected by hypertrophic GOH (a) demonstrates some villous architecture alterations and gastric foveolar metaplasia, consistent with peptic type injury. The uninvolved duodenal sections had relatively tall villi, short crypts, and many mucinous glands both above and beneath the muscularis mucosa. The areas involved with hypertrophic GOH (b) demonstrated hypertrophic oxyntic gastric mucosa with mature parietal cells.

**Figure 3:**
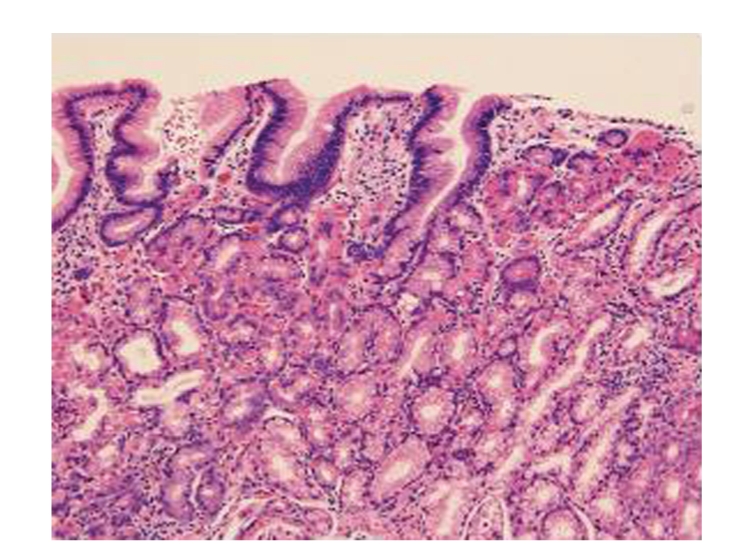
Microscopic image of hypertrophic GOH in the Duodenum. The nuclei did not display either polymorphism or pleomorphism.

Of the seven patients, with no prior gastric surgery and an intact stomach and duodenum, five were diagnosed with hypertrophic gastric oxyntic heterotopias (GOH). Methylene blue staining provided a better view of the hypertrophic, heterotopic mucosa as methylene blue was only absorbed by mature intestinal epithelium, not by gastric epithelium (Figure 1), allowing for the identification of gastric mucosa in the duodenum. Biopsies from the small bowel mucosa in the five patients with hypertrophic GOH revealed hypertrophic, well-organized, and well-differentiated gastric glands complete with chief and parietal cells (Figure 2). These duodenal biopsies with hypertrophic GOH also had portions demonstrating duodenal mucosa with multifocal gastric metaplasia of variable degrees in four patients. There was subtotal versus patchy replacement of the surface epithelium with underlying hypertrophic and mature oxyntic glands in the lamina propria seen in each of these specimens. Two of these five patients showed an immunoprofile of hyperplasia of gastrin producing endocrine cells. The deep mucosa and submucosa were invested with prominent Brunner's glands. Other specimens taken from the duodenal bulb showed areas of duodenal mucosa and adjacent oxyntic-type mucosa with overlying foveolar epithelium. Endocrine hyperplasia or neoplasia was mostly absent from the duodenal bulb. In one patient, the gastric-type mucosa contained occasional endocrine cells. On multiple biopsies, both in areas of oxyntic heterotopia and in areas with Brunner's glands, mucosa was present with nuclei displaced off to the side. The nuclei did not display either polymorphism or pleomorphism (Figure 3). The usual scant inflammatory cells are present within the lamina propria. In general, neither the gastrinoma or carcinoid tumors were present. In most cases, hypertrophic oxyntic gastric mucosa was present in the sections with normal architecture. The parietal cells were hyperplastic, hypertrophic, and displayed apical protrusions. 

Biopsies of the two patients who did not have hypertrophic GOH were conducted. Representative histology of their duodenal bulb mucosa is included (Figure 2). As was expected from gross features on endoscopic exam, histology lacking the ectopic gastric tissue that defines GOH is seen. The representative figure demonstrates some villous architecture alterations and gastric foveolar metaplasia, both of which are consistent with peptic type injury commonly seen in patients with ZES. The duodenal sections uninvolved with GOH had relatively tall villi, short crypts, and many mucinous glands both above and beneath the muscularis mucosa. *Helicobacter pylori* was not present in any of the cases. 

Injury to the mucosa identified via endoscopic biopsy in the enrolled patients demonstrated reduction in area of mucosal alteration throughout the study while on PPI therapy. In the cases of hypertorphic GOH, however, this ectopic tissue remained present despite treatment and long-term follow-up via serial endoscopy throughout the duration of the study period. 

Patients with GOH had an average serum gastrin level of 1245 pg/mL (standard deviation of 1289) and BAO of 2.92 mEq/hr (standard deviation of 4.1) versus 724 pg/mL (standard deviation of 1289) and 0.8 mEq/hr (standard deviation of 0.99) in patients without GOH. Due to limited sample size in our study of patients with this rare condition, statistical analysis utilizing student's *T*-test comparing the gastrin and acid output levels revealed no significant differences (*P* > .05) in both cases; *P* = .42, *P* = .33, respectively.

## 4. Discussion

The major symptoms of Zollinger Ellison syndrome (related to excessive gastric acid secretion) can now be well controlled with the use of proton pump inhibitors. General recommendations for the management of patients with ZES include maintaining the level of acid secretion at less then 10 mEq/hr. Patients with MEN-I syndrome, GERD (gastroesophageal reflux disease), or prior gastric acid reducing surgery should be maintained at <5 mEq/hr [19]. PPI medication was able to control acid production, evidenced by reduction in gastric acid output and symptomatic relief in all patients, but did not appear to have an effect on the hypertrophic GOH for the 5-year period of the study [20]. This ectopic gastric mucosa found in the duodenal bulb was present throughout the duration of the study, confirmed by serial biopsies performed on the patients every six months.

MEN-I status of the patients was also evaluated. ZES occurs in about one third of patients with MEN I [21]. In general, MEN I patients experience a less acute form of the disease and is associated with a genetic predisposition involving chromosome 11. It is characterized by tumors of the parathyroid, pancreas, duodenum, and anterior pituitary. Tumors in patients with MEN I are smaller, there is lower rate of metastasis, and the 20-year survival rate is much higher [22]. In addition, surgical management for patients with sporadic ZES is more promising; cure is rare following gastrinoma resection in patients with ZES and MEN I [23]. All of the patients were tested for MEN I status prior to study enrollment. This biochemical testing included ionized serum calcium and peptide hormone levels, including parathyroid hormone, gastrin, insulin, and glucagon [21]. The MEN I gene has been identified, and current recommendations are for all family members at risk to be tested annually [21]. No differences were evidenced in regards to the presence and characteristics of GOH between patients with MEN I and those without. 

Five out of the seven patients, with an intact stomach and duodenum, were positive for GOH. Patients with duodenal GOH experienced higher average baseline gastrin levels and BAO than those with no GOH in their duodenum (Table 1). The average values were not statistically significant (*P* > .05), however, largely attributable to small sample sizes and large variances. The potential trend toward higher baseline gastrin and BAO merits further study.

It has previously been reported that gastric mucosa in the duodenum with associated duodenitis was linked to the presence of *H. pylori * [24]. None of these patients, however, were diagnosed with ZES. In the present study, none of the ZES patients were infected with *H. pylori*, excluding *H. pylori * as a potential cause of the observed GOH. For these reasons, patients with *H. pylori * were excluded from the study, thus preventing the presence of a potential confounding variable. However, this does not exclude the potential for diagnosing GOH in the presence of *H. pylori*.

Lee et al. were the first to describe tumorous heterotopic gastric mucosa occurring in the small intestine in 1970 [25]. The series of fourteen cases demonstrated heterotopic mucosa in either the jejunum or ileum in seven patients. The heterotopic mucosa, however, was not identified in the duodenum of any patients in this series. The patients reported abdominal pain as being the major symptom, and ulcers were found in only three of the cases. The heterotopic mucosa was described as narrow gastric folds, similar in architecture to the duodenal GOH we identified in our patients with ZES.

Recent studies have shown gastrin acts as a growth factor in the oxyntic mucosa of mice [26]. This supports the concept that hypergastrinemia could induce ECL cell hypertrophy and hyperplasia and in our case contributes to the growth of hypertrophic GOH, making relatively large lesions visible on endoscopy and also the histopathologic findings on histology. Peghini et al. looked at hypergastrinemic diseases and their proliferative effects on ECL cells. In this study, with over one hundred patients with ZES, hypergastrinemia was correlated with ECL cell hyperplasia [27]. There have been no studies linking hypergastrinemia to gastric heterotopia in the duodenum. The fact that our patients suffer from ZES and related gastrinomas, and the trend in our data, however, suggests that an excess production of gastrin may be one component in the production of hypertrophic GOH. We hypothesize that the gastrin which induced hypertrophy may result in visibility of these lesions on endoscopy more reliably—increasing the incidence of GOH (>70% in our study) when compared to the general population with an incidence of <2%. Another possibility to consider is that these hypertrophic gastric ectopias may result in pathologic acid production in the duodenal bulb, resulting in increased incidence of postbulbar ulcers in patients with ZES—often the presenting sign. Patients enrolled in this study were on chronic acid reducing therapy from the time of diagnosis preventing acid injury of their duodenum, and thus this could not be evaluated in our subjects. Future studies utilizing endoscopic biopsy to evaluate for hypertrophic GOH on initial presentation with signs (i.e., postbulbar ulcers), symptoms, and family history consistent with gastrinoma would further elucidate the role of hypertrophic GOH.

## 5. Conclusion

The novel finding of duodenal gastric heterotopic mucosa was established in five of seven patients with ZES and an intact stomach and intestine and was likely to result from increased gastrin levels. GOH is thought to be benign in the general population occurring in about 2 percent of people. The incidence of hyptertrophic GOH, however, was 71 percent in our patients with ZES. The presence of heterotopic and hypertrophic gastric mucosa may result in gastric acid secretion in the duodenal bulb and contribute to postbulbar duodenal ulcer development, abdominal pain, and diarrhea. Furthermore, based on the biopsies taken at regular intervals during the study, this mucosa neither improved nor worsened over time with proton pump inhibitors.

## Figures and Tables

**Table 1:** Patient demographics.

Subject	Age	Sex	Race	Time since ZES diagnosed (yrs)	MEN-1 status	Gastrin level (pg/mL)	Acid output on PPI (mEq/hr)	Primary tumor site	Metastasis to liver	GOH
1	51	M	Caucasian	4	Negative	711	0.1	pancreas	No	No
2	49	M	Caucasian	12	Positive	3090	0.4	duodenum	No	**Yes**
3	61	F	Caucasian	12	Negative	2050	0.8	pancreas	No	**Yes**
4	39	M	Caucasian	25	Positive	737	1.5	pancreas	No	No
5	50	M	Caucasian	5	Negative	194	2.8	pancreas	No	**Yes**
6	59	F	Caucasian	20	Positive	121	0.6	pancreas	No	**Yes**
7	38	M	Caucasian	1	Negative	770	10	unknown	Yes	**Yes**
